# A Multifaceted Approach for Cryogenic Waste Tire Recycling

**DOI:** 10.3390/polym13152494

**Published:** 2021-07-28

**Authors:** Darkhan Yerezhep, Aliya Tychengulova, Dmitriy Sokolov, Abdurakhman Aldiyarov

**Affiliations:** Faculty of Physics and Technology, Al Farabi Kazakh National University, 71 Al-Farabi Ave., Almaty 050040, Kazakhstan; darhan_13@physics.kz (D.Y.); a.tychengulova@gmail.com (A.T.); Yasnyisokol@gmail.com (D.S.)

**Keywords:** polymer, rubber, recycling, cryoagent, liquid nitrogen, waste tire, thermal conductivity

## Abstract

One of the important aspects for degradation of the life quality is the ever increasing volume and range of industrial wastes. Polymer wastes, such as automotive tire rubber, are a source of long-term environmental pollution. This paper presents an approach to simplifying the rubber waste recycling process using cryogenic temperatures. The temperature of cryogenic treatment is ranged from 77 K to 280 K. Liquid nitrogen was used as a cryoagent for laboratory tests. Experimental and numerical studies have been carried out to determine the optimal conditions for the recycling process. Numerical studies were performed using the COMSOL Multiphysics cross-platform software. The optimal force of mechanical shock for the destruction of a tire which turned into a glassy state after cryoexposure was determined experimentally. The chemical and physical properties of the final product (crumb rubber) have been studied by scanning electron microscopy and energy dispersive X-ray spectroscopy. The analysis shows that the morphology and elemental composition of the samples remain practically unchanged, demonstrating environmental friendliness of the proposed process.

## 1. Introduction

World production of natural and synthetic rubber is growing by about 2% annually. Thus, in 2020, the total rubber production amounted to more than 25 million tons [1,2]. More than 60% of them are spent on the production of automobile tires [3]. Consequently, this causes an increase in polymer waste in the form of used tires [4]. Waste tire rubber, which needs to be disposed or recycled, is a source of long-term environmental pollution, since the degradation period of rubber in the ground is more than a hundred years. Furthermore, rubber is flammable, hazardous and is subject to long-term biodegradation, with an extremely harmful effect on the environment, in addition to poisonous substances such as benzene, xylene, styrene, toluene, etc. [5,6,7], which are released when burning.

Today, about 10 million tons of tires are annually used in the world by recycling or by other methods [2]. However, the accumulated stocks of waste tires requiring recycling or disposal are about 100 million tons. These data indicate a large volume of potentially valuable raw materials that require processing for further use [8,9]. According to preliminary evaluation, only about 10% of waste tires can be recovered, but this only delays the moment when they need to be disposed or recycled [10,11].

Despite its enormous scale, the problem of waste tire rubber recycling is solvable, and there are many ways to do it [12,13,14,15,16,17]. In principle, all known methods of waste tire rubber recycling can be divided into two main groups: physical and chemical. Chemical processing methods lead to irreversible chemical changes not only in rubber, but also in the constituent substances (vulcanizers, softeners, plasticizers, etc.). These methods are carried out at high temperatures resulting in material destruction [18,19,20,21,22]. Despite the fact that chemical methods of waste rubber recycling make it possible to obtain valuable products and heat, such use is not efficient enough, since it does not allow preserving the original polymer materials.

Physical methods of processing automobile tires include various methods of grinding aiming to obtain rubber crumb, which most fully preserves the properties of rubber [23,24]. The physical process of rubber grinding is rather complicated, since, due to its high elastic properties, the energy consumed for destruction is spent mostly on mechanical losses. The efficiency of mechanical rubber grinding is largely dependent on temperature and load application rate. If the grinding process occurs at a temperature below the glass transition temperature of rubber, then its deformations are small, and the destruction is brittle [25,26]. At the same time, it can be assumed that among physical methods, the low-temperature method is very promising [27,28].

The cryogenic method has the following advantages over the room temperature methods (i.e., when the rubber is in an elastic state): significantly less energy consumption for mechanical grinding; exclusion of fire and explosion hazard; production of a finely dispersed rubber powder with a particle size of up to 100 microns; elimination of environmental pollution [28]. In general, the effectiveness of cryogenic tire grinding is a consequence of: (a) bond weakening between metal cord and rubber at low temperatures, which leads to partial separation of rubber from metal; (b) sharp decrease in the elasticity of rubber and its brittle destruction even at minor deformations [27].

Bibliographic research has shown that it has not yet been clearly defined which of these methods is more efficient in terms of environmental impact, energy consumption and product application [29,30]. Articles [31,32] mentioned the unpromising nature of the cryogenic method, and also considered such issues as the method application and the economic benefit from cryogenic processing of automobile tires. The authors in [33,34] concluded that the cryogenic method is not economically feasible. These conclusions are mainly based on the cost of the primary material for cold production—liquid nitrogen. The market value of liquid nitrogen in 1980s was 4.05 $/kg, but today the commercial price varies from 0.2 to 1 $/kg, depending on the technical and economic capabilities of the region and the manufacturer. It should be noted that to assess the economic feasibility of using the cryogenic method, it is necessary to consider the prime cost of the refrigerant. As an example, we estimated the primary cost of one kilogram of liquid nitrogen produced in the laboratory of cryophysics and cryotechnology of Al Farabi Kazakh National University, which was 0.1 $/kg. Obviously on an industrial scale this price is expected to be much lower (≈0.05 $/kg). In addition, an indicator of the liquid nitrogen availability is the fact that it has become widely used in modern companies, ranging from cosmetology to industry [35,36,37,38,39,40,41,42]. Moreover, the use of air turbine refrigeration machines can reduce the cost of cold production by 3−4 times, and specific energy consumption by 2−3 times compared with the use of liquid nitrogen [43].

Thus, the simplification of rubber waste recycling using cryotemperatures is the subject of the present work aiming to reduce the required energy consumption. The object of the study is waste automobile tires. The temperature range of cryogenic treatment varied from 77 K to 280 K. The optimal force of mechanical shock for the tire destruction which turned into glassy state after cryoexposure was determined experimentally. The chemical and physical properties of the crumb rubber have been studied by scanning electron microscopy and energy dispersive X-ray spectroscopy.

## 2. Materials and Methods

### 2.1. Materials

The object of the present research is automobile tires manufactured according to the standard “GOST 4754-97 (Pneumatic tires for passenger cars, trailers for them, light-duty trucks and buses of especially small capacity Specifications)” (“ISO 10191: 2010” Passenger car tires—Verifying tire capabilities—Laboratory test methods). In the experiment, one-piece waste tires were used to determine the parameters of mechanical impact.

### 2.2. Measurement of Thermal Conductivity of Samples

Knowledge of the thermophysical properties of polymeric materials is important in many industries and technological development. The ability to identify the temperature dependence of polymer properties in a wide temperature range, including low temperatures, can play a particularly decisive role [25,44,45].

For computer simulations of the cooling process of a one-piece automobile tire, we used experimental data of thermal conductivity for a rubber sample. These experimental data were presented in our earlier work [46] in a graphical form. In this work, experimental measurements of the thermal conductivity of various samples were carried out in the low-temperature range from 95 K to 275 K. The samples were tire fragments consisting of pure rubber, rubber with nylon and metal cords. The data in [46] were used only for pure rubber and are shown in Table 1.

As can be seen from Table 1, the value of thermal conductivity coefficient is maximum at 215 K, equal to 0.331 W/m · K. For the sample under investigation, this temperature is the glass transition temperature, i.e., *T*g = 215 K, the temperature at which there is a phase transition from an elastic to a brittle glassy structure occurs [46,47,48].

### 2.3. Mechanical Characterization

**Figure 1:**
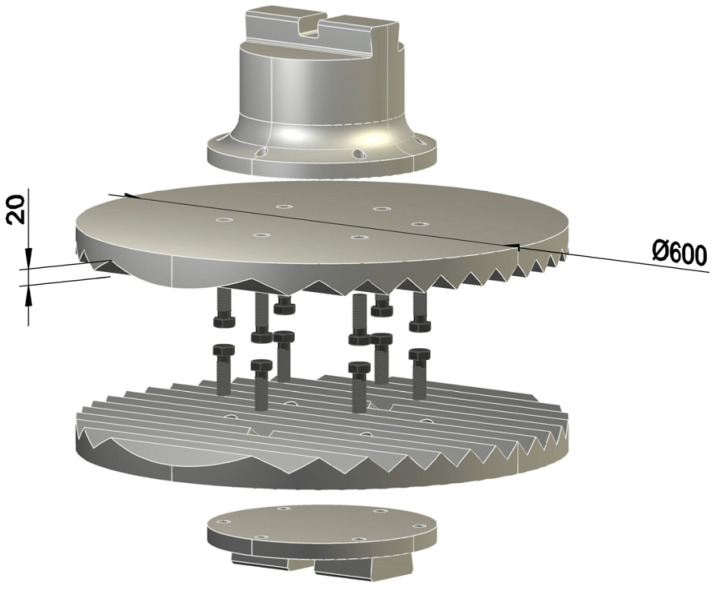
Grooved contact part of the sledgehammer.

To calculate the destruction force of mechanical impact on a glassy tire we performed several experiments using a sledgehammer. The experiment involved a pneumatic sledgehammer of type MA4129A (LLC South Ural Mechanical Plant, Kuvandyk, Russia) with dimensions of 830 × 1560 mm^2^ and a nominal mass of falling parts 80 kg. The maximum speed of falling parts at the moment of impact (theoretical) was 6.16 m/s. Minimum impact energy, not less than 155 kgf·m. The longest tup stroke was 365 mm. The rated power of the machine is 7.5 kW. The standard hammer strikers were replaced with profiled punches and dies (see Figure 1), especially made for the optimal sizes of automobile tires, in order to apply the mechanical shock entirely to the cooled tire. 

The calculation of the impact force was made according to the following considerations: a hammer of mass *m* strikes a frozen sample with a speed *υ*_1_ and rebounds after an impact with a speed *υ*_2_. If we take into account that the impulse of the hammer *p*_2_ after the impact is directed oppositely to the initial impulse *p*_1_, then the change in impulse will be Δ*p = m* (*υ*_1_ *+* *υ*_2_). The expression for the force impulse is written as: *F*Δ*t =* Δ*p*. The average hammer force acting on the sample during the impact was determined, assuming the contact time of the hammer with sample equal to Δ*t* = 10^−3^ s.

### 2.4. Mathematical Model

The COMSOL Multiphysics program was used to simulate and calculate the optimal cooling time for a car tire. The main task of the modeling is the solution of non-stationary thermal fields described by the heat conduction equation, which belongs to the parabolic equations of the second order. The numerical experiment was performed using the “Heat Transfer” and “Computational Fluid Dynamics” modules of COMSOL Multiphysics. The Heat Transfer and Computational Fluid Dynamics (CFD) modules extend the capabilities of the COMSOL Multiphysics numerical simulation environment for the numerical analysis of systems in which hydrodynamic processes are accompanied by various physical phenomena [49].

Waste tire rubber and liquid nitrogen are modeled as two different computational domains, accompanied by the phenomenon of conjugate heat transfer. For each region, the corresponding differential heat conduction equations are used. These equations are shown below:

Heat transfer in the liquid nitrogen model:

(1) $$\rho_{f}c_{f}\frac{\partial T}{\partial t}+\nabla \cdot (-\lambda_{f}\nabla T)+\rho_{f}c_{f}T\nabla u=0$$

where *ρ_f_* is the liquid density, *c_f_* is the specific heat of liquid, *λ_f_* is the thermal conductivity of liquid, and **u** is the velocity field. Equation (1) represents a homogeneous partial differential equation.

Heat transfer in the simulated tire:

Due to the absence of any mass flow **u** is taken to be zero in the simulated tire. Therefore, the basic heat transfer equation takes the following form:

(2) $$\rho_{s}c_{s}\frac{\partial T}{\partial t}+\nabla \cdot (-\lambda_{s}\nabla T)=0$$

where *ρ_s_* is the density of the tire material, *c_s_* is the specific heat capacity of the tire, *λ_s_* is the thermal conductivity of the tire.

The tire model consists of two elements—a rubber component and a metal (cord). The key point in the heat transfer process at the boundary of these elements is the use of the standard finite element method with matched grid cells with common nodal points at which the solution to the problem is calculated. Grid elements that have common nodes and the continuity condition of the sought field variables are automatically defined. The balance of heat fluxes between elements belonging to different regions is adjusted automatically. Since the elements have common nodes, the temperature fields in these nodes are continuous. The fulfillment of the continuity condition is ensured by duplicating Equation (2) with the corresponding properties.

The CFD module includes special physics interfaces for simulating conjugate heat transfer between a fluid and a solid. For the laminar mode of the convective fluid flow, the condition of temperature continuity at the solid-liquid interface is used (this is the standard setting in non-isothermal flow interfaces). Non-isothermal laminar flow interfaces and conjugate heat transfer are required to conserve energy, mass, and momentum in fluid, and to conserve energy in solid. When solving this problem in COMSOL Multiphysics, the predefined Laminar Flow multiphysics interface in the Conjugate Heat Transfer branch automatically adds the Heat Transfer in Solids and Fluids interface. It is used to simulate heat transfer based on heat conduction and convection.

The following initial and boundary conditions are accepted in the computer model: *p* = 0—constant pressure at the upper boundary, **u** *=* **n***U*_0_ = 0—on the symmetry axis of the element, **u** *=* **u***_f_finite_*—on the lower boundary, **u** = 0—on all other boundaries, initial ambient temperature is 77 K, simulating the temperature of liquid nitrogen.

**Figure 2:**
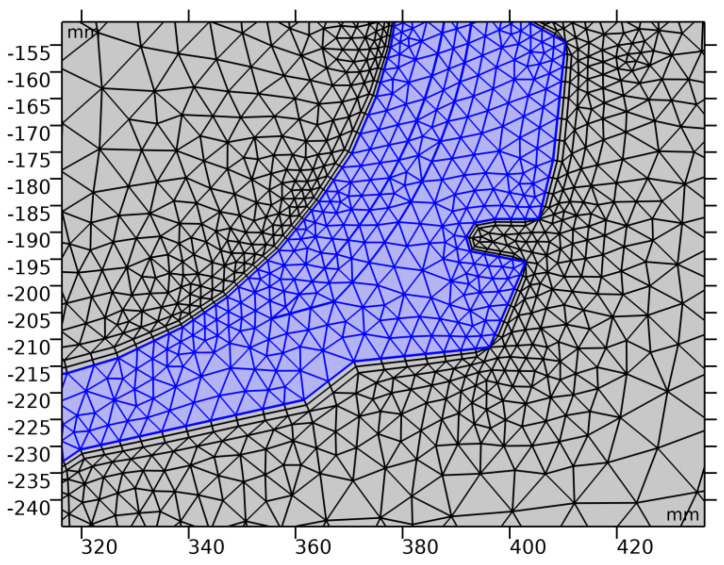
Fragment of the grid model for COMSOL computations.

The model grid was built choosing the optimal number of cells with a minimum loss of computational resolution. In this regard, the model was divided into 67,455 finite elements (triangles). The minimum grid element size is 0.1 mm, and the maximum is 23.5 mm. A fragment of the grid model is shown in Figure 2.

### 2.5. Evaluation of Model Adequacy

The evaluation of the computer model adequacy was performed by comparing the simulation results with experimental data obtained by registering the temperature change of a rubber sample during its cooling by liquid nitrogen. The sample has a cubic shape with geometric dimensions of 1.5 × 1.5 × 1.5 cm^3^, and it was derived from a massive piece of tire. A thermocouple (type T) was used as a thermal sensor and was inserted into the center of the rubber sample using a thin tubular catheter.

**Figure 3:**
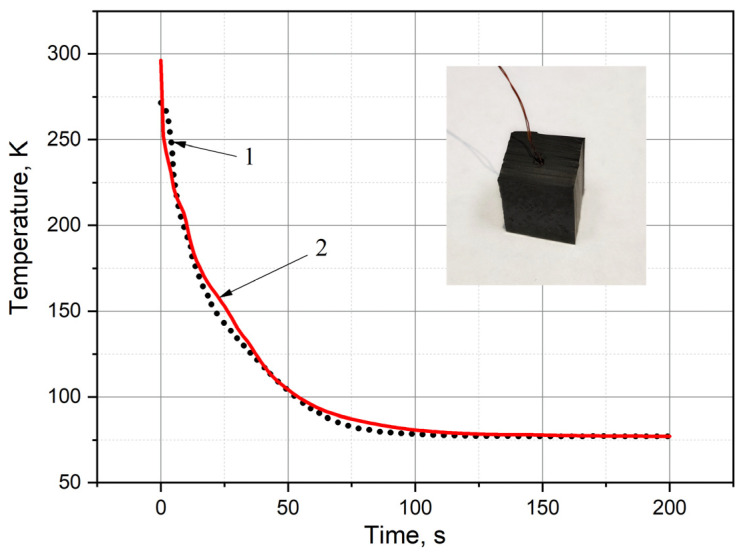
Comparison of the experimental and simulation data. 1—experiment, 2—model.

Furthermore, the sample was cooled by immersion in liquid nitrogen with simultaneous recording of its temperature change over time in an automatic mode using a PC. The process of recording the temperature sensor readings was carried out from the start of cooling until the sample was taken to the temperature of liquid nitrogen. The initial sample cooling temperature was room temperature (293 K). The experimental results (curve 1) are shown in Figure 3.

The above experiment was simulated using the COMSOL Multiphysics environment with detailed description given in Section 2.4. Geometry in the computer model was taken in accordance with the dimensions of the experimental sample. The temperature range was set from 293 K to 77 K. The results of numerical experiment in comparison with the experimental data are shown by curve 2 in Figure 3. It can be seen from Figure 3 that the results of numerical simulation and experiment are in good agreement, and the deviation of the experimental data from numerical simulation curve does not exceed 10%.

### 2.6. Scanning Electron Microscope and Energy Dispersive X-ray Spectroscopy

The morphology and elemental composition of the crumbs were studied by scanning electron microscopy (Quanta 200i 3D, FEI Company, Hillsboro, OR, USA) and energy dispersive X-ray spectroscopy (MicroXRF Analysis Report, EDAX int, Berwyn, PA, USA).

## 3. Results and Discussion

### 3.1. Evaluation of the Mechanical Impact Force

It is known that, depending on the temperature, natural rubbers can reside in one of the three states: glassy, highly elastic, and viscous-flow [50,51]. The elasticity of rubbers can be completely lost depending on the degree of cooling [52]. If the temperature dependence of elasticity is characterized by mechanical properties, then taking the value of deformation at a given strain and temperature as a characteristic of the rubber state, it is possible to obtain thermomechanical data. This, in turn, can make it possible to compare the value of deformation at different temperatures, and also to find the glass transition temperature corresponding to the transition of rubber to a brittle glassy state [53]. Glass transition depends not only on the temperature, but also on the nature of the mechanical load. Thus, under static loads or dynamic loads of low frequency, the glass transition temperature is lower than under dynamic loads of high frequency [54]. From this perspective, it is clear that the most accurate measurement of the cooling depth of samples and mechanical effect after freezing are necessary to optimize the low-temperature method for rubber waste recycling. As known, the duration of cooling depends on the mass, geometric parameters and thermophysical properties of waste tires [28].

**Figure 4:**
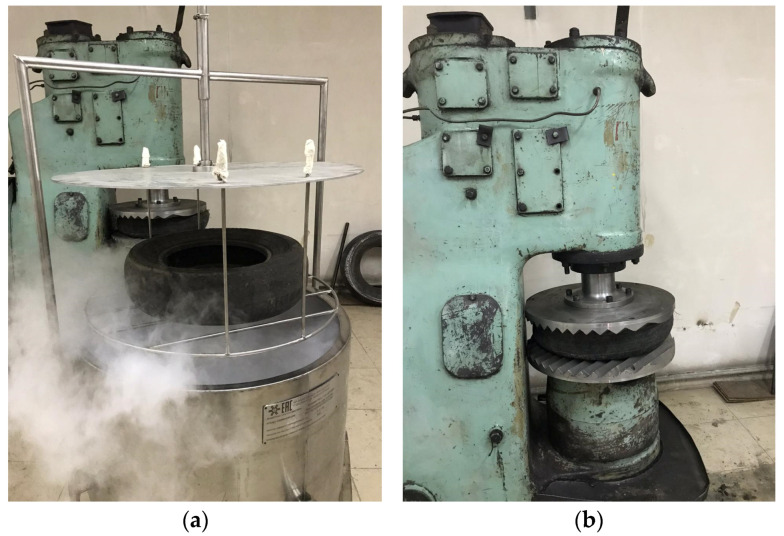
Cooling process of automobile tire in liquid nitrogen (**a**) and mechanical impact on frozen tire (**b**).

The experiment on cryogenc grinding of car tires was carried out in few steps. On the first step cooling of the waste tire was done by complete immersion in special vessel, made for this purpose (Figure 4a). The time of immersion was determined starting from the immersion moment up to the termination of intense boiling of liquid nitrogen. The cooling time, depending on the mass and average thickness of the tire walls, varied from 200 s to 300 s. Here, the degree of cooling is very important, since it is necessary to have a temperature margin of brittleness, which can be spent due to the heat of dissipation of a mechanical shock. 

Further the frozen waste tire was placed under the hammer (Figure 4b) and subjected to mechanical stress (impact) in a single mode. The strength of the mechanical impact and the number of blows required to completely destroy the tire were determined by varying the speed of the hammer blow. According to the results of tire destruction, the optimal force of one impact was calculated. If necessary, the number of blows can be increased to achieve the required degree of separation of the metal cord and nylon fibers.

The calculation of the force of mechanical impact was carried out experimentally as follows: a hammer of mass m = 80 kg strikes a frozen tire with a speed υ_1_ ≈ 2.5 m/s, and stops after the impact without rebound, i.e., with υ_2_ ≈ 0 m/s, spending all the impact energy on the destruction of an embrittled tire. Furthermore, making simple mathematical calculations, according to the considerations of Section 2.3, the required value of the force of mechanical shock was determined. 

After the mechanical impact, the crushed rubber crumbs were sorted by size depending on the strength of the impact. The metal and nylon cord elements are almost completely separated from the rubber component (up to 95%), which can significantly reduce further costs for products recycling by other combined methods. It is important to note that this fact is the main advantage of the cryogenic method of recycling waste tires [28,55].

**Figure 5:**
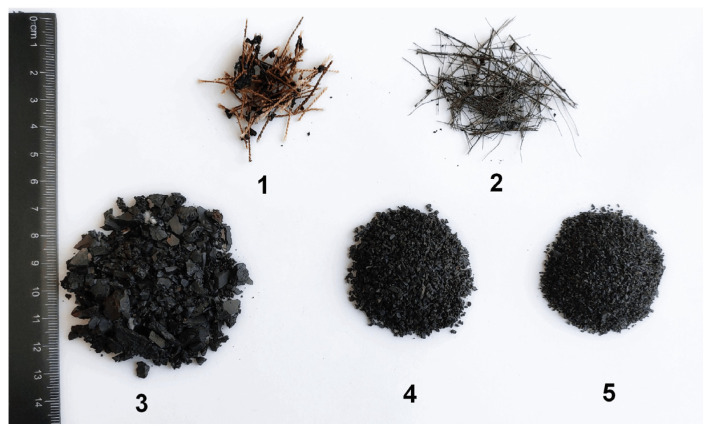
Typical products of cryogrinding. 1—nylon cord, 2—metal cord, 3—large size fraction of rubber crumb (1–5 mm), 4—mean size fraction (1–2 mm), 5—small size fraction (0.1–1 mm).

According to preliminary estimates, in one hammer blow, up to 80% of rubber contained in the tire passes into the crumb, with 50% of the crumb with dimensions from 1.25 to 20.00 mm and 25% from 0.10 to 1.25 mm. The evaluation showed that the force with an approximate value of ≈200 kN is a satisfactory result for the destruction of the frozen tire. Figure 5 shows samples of rubber crumb of various fractions with separated metal rods.

### 3.2. Computer Simulation

Numerical experiment of the tire rubber cooling process was performed using axisymmetric 2D modeling. Many physics interfaces are available in axisymmetric versions and consider the axial symmetry. This allows solving a 2D problem in plane instead of a full 3D model problem, which can significantly save machine memory and computation time. After solving the problem in plane, we can get the result in a three-dimensional form by rotating the two-dimensional axisymmetric solution. 

**Figure 6:**
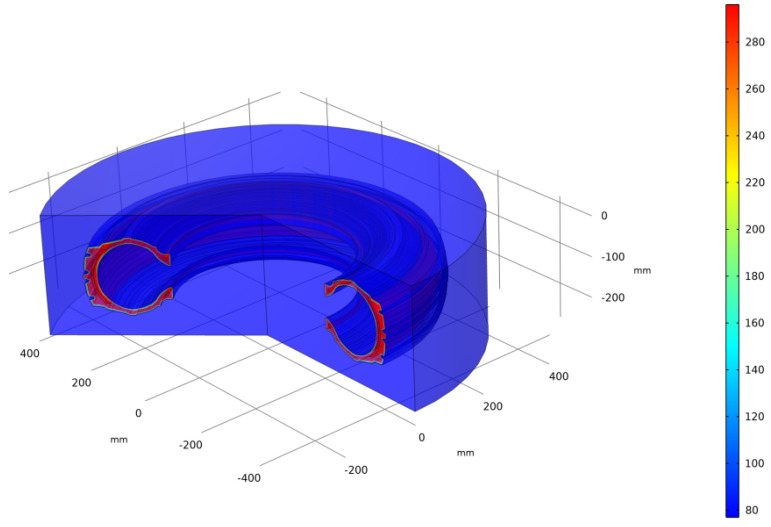
Computer model illustration.

The simulated model of car tire has the following geometrical dimensions: width—185 mm; tire profile—75%; landing diameter—13 inches. The sketch of the 3D model, which is shown by rotating the cross-section of the 2D model around the axis located at the distance of tire radius, is shown in Figure 6.

Experimental data on the temperature dependence of the rubber thermal conductivity, given in Table 1, were used as the values of thermal conductivity *λ_s_* of the simulated tire, and the values of density *ρ_s_* and heat capacity *c_s_* were taken from tabular data [56,57]. For the coolant and the metal cord, the values of these physical quantities (*ρ_f_*, *c_f_*, *λ_f_*) were also taken from the tabular data [58,59].

The main pre-definitions of the COMSOL Multiphysics software package for numerical experiments are given in Section 2.4. To be clear, we note that the initial tire temperature was taken equal to T = 293 K, and the temperature of the surrounding liquid medium was set equal to T = 77 K (T = *const*). The process of conjugate heat transfer is carried out between two subsystems, i.e., heat exchange occurs between solid (tire) and liquid (liquid nitrogen).

Please note that the calculation of the heat transfer coefficient which characterizes the intensity of heat transfer on the surface of the simulated tire is essential. It is known that the use of the similarity equation for the dimensionless Nusselt number is a recognized method for the calculation of heat transfer coefficient. These equations make it easy to calculate the heat transfer coefficient for different heat transfer conditions. However, this method is used only for objects of regular geometric shapes, for example, flat surfaces, cylinders and spheres. If the heat transfer surface has a more complex shape, as in the present work, then the heat transfer coefficient is calculated by simulating the conjugate heat transfer. Thus, the present model was numerically solved in COMSOL Multiphysics using the Conjugate Heat Transfer interface, which allows us to calculate the flow and temperature fields in liquid. Based on the simulation results, the program calculates the heat flux density with reference to the corresponding built-in post-processing variable. Furthermore, by dividing the reported value of the heat flux density by the temperature difference (*Ts-T_L_*), the heat transfer coefficient can be found. 

**Figure 7:**
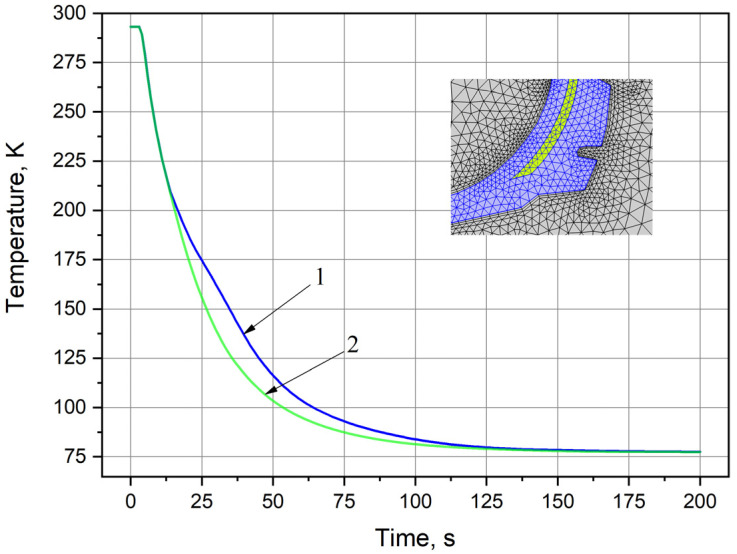
Tire cooling graph. 1—average temperature change in grid nodes for the entire model cross-section; 2—average temperature change in grid nodes for the cross-section of the metal cord.

Figure 7 presents the model calculation results. The graph shows the decrease in average temperature of the simulated tire over time. Average temperature is the temperature of the grid nodes divided by the number of these nodes in the selected object. Curve 1 shows the decrease in average temperature of the model rubber component. In addition, curve 2 shows similar decrease for only the metal cord. As expected, the change over time in average temperatures of the subsystems from 300 K to 77 K occurs according to the exponential law (Newton’s cooling law [60,61]). It can be seen from Figure 7 that the decline of curve 2 is faster than curve 1. This is explained by the difference in properties of the subsystems set for the simulated materials and the geometric locations of the grid nodes, in which the temperature changes over time are determined. It is clear that the values of the thermal conductivity coefficients of rubbers are two orders of magnitude lower than those of metals [62,63]. Therefore, determination of the average temperature for simulated part of the rubber with the cord at each moment of time occurs faster than the average temperature of the entire model area. In addition, the cord is not in the center, but closer to the border surface. If we assume that cryogenic treatment is performed on both sides of the interface, then the relatively rapid cooling of the rubber with cord in comparison with the entire model is quite obvious. Thus, the simulation is based on the cooling process of investigated object from the known initial temperature to the glass transition temperature [47] and further to temperature 77 K for cold margin at a given time. The duration of time is determined by the series of average values between time t = 0 and the estimated time. These values were calculated in accordance with the given thermal conductivity and temperature of the object using the calculation of moving average. The calculated time value is compared with the experimental cooling time presented in Figure 3. Good convergence between two values can be seen.

**Figure 8:**
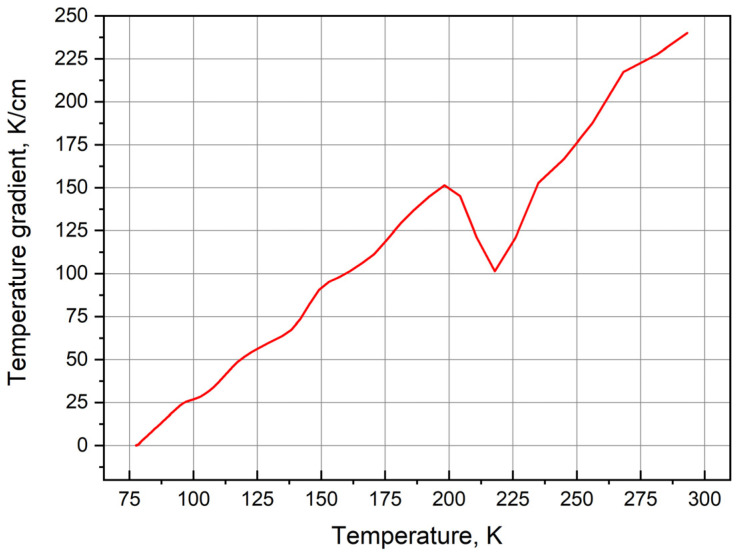
Temperature dependence of temperature gradient.

Obviously, the thermal conductivity coefficient describes the relationship between the heat flux vector **q** and the temperature gradient Δ*T*, which are included in the Fourier law equation. Since the present model uses the experimental coefficients of rubber thermal conductivity in the low-temperature range (95–275 K), we plot the dependence of temperature gradient on the average temperature at two measured points in order to further check the model adequacy with experiment. In the COMSOL Multiphysics software package, the temperature gradient was determined between two points chosen arbitrarily according to the condition that they lie along the axis normal to the surface of the isotherm. This dependence is shown in Figure 8. In general, the temperature gradient values linearly decrease with decreasing temperature. However, in the vicinity of 220 K, the linear dependence of the temperature gradient undergoes a sharp decline. This is explained by the fact that in the vicinity of this temperature (220 K), the phase transition from the elastic to the glassy state occurs in rubber [46,47,48,64].

### 3.3. Physical and Chemical Properties

Morphology and chemical composition of crumb rubber were studied using a scanning electron microscope (Quanta 200i 3D, FEI Company, USA) and energy dispersive X-ray spectroscopy (MicroXRF Analysis Report, EDAX int, USA). The purpose of these studies was to understand the physical and chemical properties of the crumb rubber after cryodestruction and to formulate recommendations for further use of recycled rubber crumbs.

**Figure 9:**
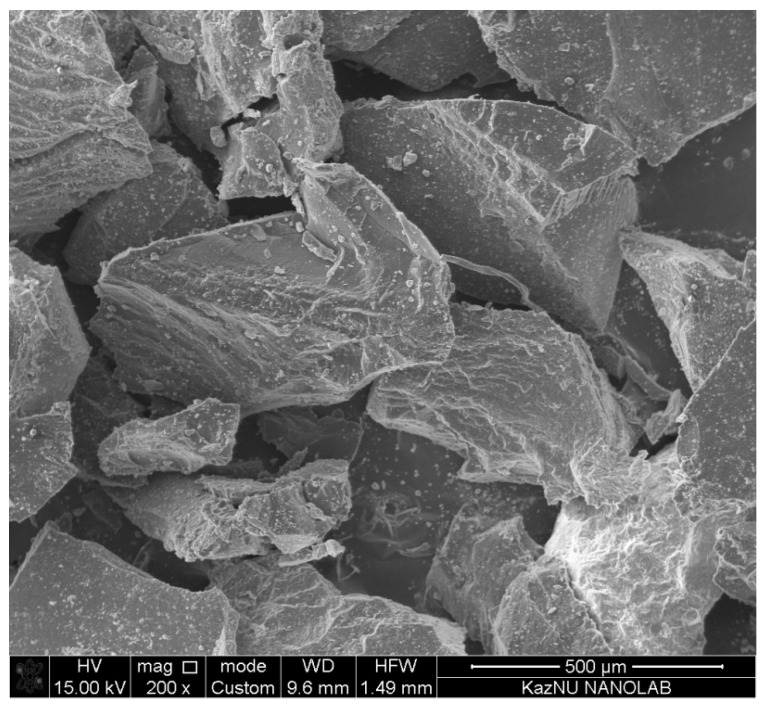
SEM—cryodestructed crumb rubber sample illustration.

Figure 9 shows the results of microscopic examination of the rubber crumbs. It can be seen that rubber particles have a smooth surface, characteristic of large particles, without microcracks and pores. The ability to produce rubber crumbs without the complex surface (microcracks and pores) is one of the advantages of the cryogenic method of polymers destruction. It is known that fine particles of any solid substance with a complex surface efficiently adsorb ambient gases, particularly atmospheric gaseous oxygen. Adsorbed atmospheric oxygen can lead to rapid oxidation of the polymer, therefore shortening its shelf life. In addition, the oxygenated polymeric material can be extremely flammable.

**Figure 10:**
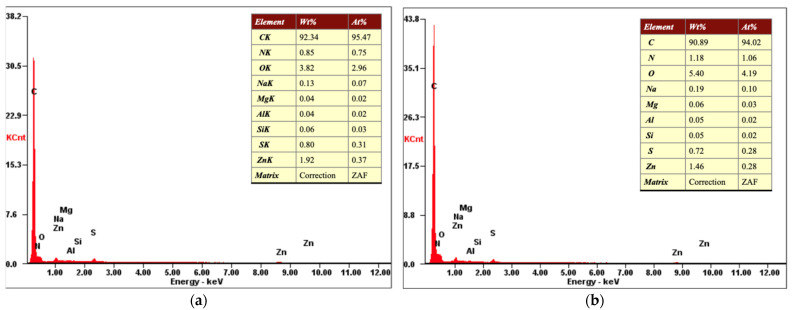
Elemental composition of crumb rubber before (**a**) and after (**b**) cryogenic destruction.

Figure 10 shows the results of the elemental composition analysis for the tire sample before (a) and after (b) cryodestruction using energy dispersive X-ray spectroscopy (MicroXRF Analysis Report, EDAX int, Berwyn, PA, USA). A wide range of chemical compounds can be observed in the waste tire rubber, such as natural rubber, SBR (styrene butadiene rubber), butadiene rubber, etc. A large number of other chemicals are also added to tire rubber, such as: vulcanizing agents (sulfur and sulfur compounds), catalysts, inhibitors, pigments, etc. Vulcanizing agents and various additives are used in tires to create a cross-linked structure and provide resistance to various physical and chemical influences [65,66]. Other micro-additives such as calcium, magnesium, sodium, potassium, chloride, etc. are added to improve the mechanical performance. The complex composition of car tires requires performing a chemical content analysis for the presence of toxic elements harmful to the environment. It is also important to accurately identify the characteristics of the recycling products and by-products to assess the environmental footprint of the processing technique. 

Thus, elemental composition of the crumb rubber after cryodestruction showed no significant difference with the energy dispersive analysis of the original tire sample before the cryogenic treatment.

## 4. Conclusions and Recommendations

The annual reports of many international companies and scientific journals show that the growth rate of rubber and industrial rubber goods production is increasing annually. Consequently, the growth rates of waste rubber-containing products is also increasing. More than half of this waste consists of the waste tire rubber of various sizes. Therefore, waste tires are a pressing hazard, affecting environment on a global scale. Unfortunately, industrial development in its present form is accompanied by violation of the ecological balance as a result of human impact on the nature.

Based on the results of the present work, the following brief conclusions can be drawn:

Mechanically stressed glass tire is cooled by full immersion in liquid nitrogen. The duration of tire cooling was determined experimentally. Analysis of the samples on the mechanical impact force showed that the force with approximate value 200 kN is a satisfactory result for the destruction of tire to crumb rubber. 

A numerical model of the car tire cooling process showed satisfactory results, correlating with the experiment. These results can be useful in the design of cryogenic reactors for solid household polymer waste recycling.

The results of morphology and chemical composition studies of crumb rubber after cryodestruction can be used when crumb rubber is applied as a filler in various structural materials.

## Figures and Tables

**Table 1:** Temperature dependence of thermal conductivity of a rubber sample.

*T*, K	λ, W/m·K
95	0.142 ± 0.003
105	0.187 ± 0.017
115	0.166 ± 0.005
125	0.177 ± 0.005
135	0.185 ± 0.009
145	0.142 ± 0.006
155	0.164 ± 0.005
165	0.174 ± 0.011
175	0.155 ± 0.011
185	0.157 ± 0.006
195	0.154 ± 0.004
205	0.236 ± 0.005
215	0.331 ± 0.012
225	0.184 ± 0.006
235	0.209 ± 0.011
245	0.218 ± 0.003
255	0.173 ± 0.001
265	0.148 ± 0.001
275	0.124 ± 0.002

## Data Availability

The data used to support the findings of this study are available from the corresponding author upon request.
